# Effects of Pharmacist Intervention on the Utilization of Vancomycin in a Teaching Hospital

**Published:** 2015

**Authors:** Maria Tavakoli-Ardakani, Samaneh Ghassemi, Afshin Mohammad Alizadeh, Jamshid Salamzadeh, Mojtaba Ghadiani, Sara Ghassemi

**Affiliations:** a *Department of Clinical Pharmacy, School of Pharmacy and Pharmaceutical Sciences Research Center, Shahid Beheshti University of Medical Sciences, Tehran, Iran*; b *Student Research Committee, School of Pharmacy, Shahid Beheshti University of Medical Sciences, Tehran, Iran. *; c *Division of Infectious Diseases, Taleghani Hospital, Shahid Beheshti University of Medical Sciences, Tehran, Iran. *; d *Department of Hematology-Oncology, Taleghani Hospital, Shahid Beheshti University of Medical Sciences, Tehran, Iran.*

**Keywords:** Pharmacist intervention, Vancomycin, Drug use evaluation, Guidelines, Antibiotic use

## Abstract

In order to investigate the effect of pharmacist intervention on vancomycin use, this study was performed on all patients receiving vancomycin in the intensive care unit (ICU) and hematology-oncology ward of Taleghani Educational Hospital in Tehran, Iran. Vancomycin use was assessed during a pre- and post-intervention period in accordance with the Center of Disease Control and prevention (CDC) and Infectious Diseases Society of America (IDSA) guidelines. Following the intervention, there was a significant change in appropriate initiation of vancomycin (*P *= 0.009) and no significant improvement was observed in adequate dosage and the duration of therapy (*P *= 0.15 and *P *= 0.54 respectively); however, informing the physician resulted in discontinuation of the drug in 50% of inappropriate cases and vancomycin dosage was adjustedin 31% of cases. Temperature charts, culture results and pre-treatment CBC tests changed significantly (*P *= 0.02, P = 0.009 and *P *= 0.04 respectively). The rate of infusion related adverse drug reactions did not decrease significantly (*P *= 0.06); yet in 100% of patients, these reactions were resolved after notifying the nursing team. After pharmacist intervention,vancomycin use improved in some aspects. A significant improvement in appropriate initiation of therapy was observed; however, treatments continued despite negative cultures. It is necessary to optimize the use of vancomycin by performing more educational interventions.

## Introduction

Over the past two decades, development of multiple resistances to antibiotics among gram positive organisms has raised concern (1, 2).Strains of *Staphylococcus aureus* resistant to methicillin (MRSA) are considered one of the main causes of hospital acquired infections and acquired resistance to conventional antibiotics makes their treatment difficult (3).Infections caused by MRSA and Staphylococcus coagulase-negative have increased and leftvancomycin as the antibiotic of choice in the treatment of these infections (4, 5, 6).

Unfortunately,inappropriate use of vancomycin has increased the resistance among gram positive cocci and is considered to be a great risk factor of development and colonization with vancomycin resistant enterococci (VRE) (2, 7-14).Other risk factors associated with VRE colonization or infection are admission to an intensive care unit (ICU), hematology-oncology or transplant wards, presence of an indwelling urinary or central venous catheter and prolonged hospital stay (12, 15-20).

From 1989 to 1993, reported nosocomial enterococcal infections caused by VRE to the Centers for Disease Control and Prevention (CDC) increased from 0.3% to 7.9% (21). Also, studies showed that VRE infections occurred more frequently in large hospitals (≥ 200 beds) and university affiliated hospitals (13, 22).

In order to promote vancomycin use, the Hospital Infection Control Practices Advisory Committee (HICPAC) of the Centers for Disease Control and Prevention has published recommendations for preventing the spread of VRE (Table 1) (1). The purpose of the present study was to evaluate the appropriateness of vancomycin use before and after pharmacist intervention according to HICPAC and Infectious Diseases Society of America (IDSA) guidelines in the hematology-oncology ward and the intensive care unit in a tertiary teaching hospital in Iran (1, 23).

**Table 1:** Published criteria by the CDC for vancomycin use (1).

Vancomycin Use
Appropriate Serious infections caused by beta-lactam resistant gram-positive microorganisms Infections caused by gram-positive microorganism in patients allergic to beta-lactam antimicrobials Antibiotic-associated colitis that fails to respond to metronidazole therapy or is severe and potentially life-threatening Prophylaxis, as recommended by the American Heart Association, for endocarditis following certain procedures in high risk patients Surgical prophylaxis, with prosthesis implant, in institutions with high rates of infections caused by MRSA or methicillin-resistant *Staphylococcus epidermidis* Inappropriate Routine surgical prophylaxis other than in patients with a life threatening allergy to beta-lactam antibiotics Empiric antimicrobial therapy for a febrile neutropenic patient, unless strong evidence is present of an infection caused by gram-positive microorganisms and the prevalence of infections caused by MRSA in the hospital is substantial Treatment of a single blood culture for coagulase-negative Staphylococcus if other blood cultures collected simultaneously are negative Continued empiric use in patients whose cultures are negative for beta-lactam-resistant gram-positive microorganisms Systemic or local prophylaxis for infection or colonization of indwelling central or peripheral intravascular catheters Selective decontamination of the gastrointestinal tract Eradication of MRSA colonization Primary treatment of antibiotic-associated colitis Routine prophylaxis for very low-birthweight infants Topical application or irrigation of vancomycin solution Treatment (chosen for dosing convenience) of infections caused by beta-lactam-sensitive gram-positive microorganisms in patients with renal failure Routine prophylaxis for patients on continuous ambulatory peritoneal dialysis or hemodialysis

MRSA: methicillin resistant *Staphylococcus aureus*

## Experimental

***Methods***

The present study was approved by Ethical Committee of the Shahid Beheshti University of Medical Sciencesand conducted at the intensive care unit and hematology-oncology ward of Taleghani Teaching Hospital in Tehran, Iran, between January 21, 2011 and January 21, 2012. All the patients prescribed intravenous vancomycin were enrolled in the study. We evaluated vancomycin use at two intervals: at baseline and during pharmacist intervention.

The medical charts and laboratory data of patients receiving vancomycin were reviewed by a pharmacist. Also further data was collected from the patients and from the medical staff. Extracted data included demographics, indication, dosing regimen, rate and duration of administration, culture and sensitivity results, medication history, adverse drug reactions, white blood cells (WBC) counts, serum creatinine, urine analysis and blood urea nitrogen. To indicate the appropriateness, vancomycin use was assessed according to the criteria published by Hospital Infection Control Practices Advisory Committee of the CDC and guidelines published by IDSA.

The major aspects of vancomycin misuse were clarified following the primary evaluation of vancomycin administration. The intervention began on July 23, 2011, and evaluated the same parameters. The two phases of our study were performed under the same circumstances.We monitored accurately each patient for whom vancomycin was prescribed. Based on the guidelines and consultations with the infection diseases specialist, we determined the accuracy of each treatment. Whenever vancomycin use was not in accordance with the guidelines, pharmacist contacted the physicians, informing them about inappropriate vancomycin use. If the previous strategy still continued despite the intervention, a discussion with physician was considered.

Data analysis was done by chi-square (χ^2^) or Fisher’s exact tests and significance was defined as a p-value lower than 0.05.

## Results and Discussion

***Pre-intervention data***

During the first monitoring period, a total of 77 patients were evaluated. The most common reason for vancomycin use was fever and neutropenia (16.35%) (Table 2). Initiation of therapy was compatible with the guidelines in 38.96% of patients and duration of therapy was considered appropriate in 83.33% of patients for whom vancomycin was initiated correctly. Only 54.55% of cases received an appropriate dosing regimen based on age, weight and creatinine clearance calculated by the Cockcroft-Gault equation.

**Table 2:** Characteristics of 159 hospitalized patients receiving vancomycin at Taleghani Teaching Hospital

Variable	Before intervention: n (and %)	Post-intervention n (and %)
Sex		
Male	41 (53.25)	51 (62.2)
Female	36 (46.75)	31 (37.8)
Mean age ± SD	44 ± 16.7	49 ± 20.24
Ward		
ICU	15 (19.48)	29 (35.37)
Hematology-Oncology	62 (80.52)	53 (64.63)
Diagnosis		
Fever and neutropenia	26 (16.35)	24 (15.09)
Fever	17 (10.69)	13 (8.18)
Pneumonia	9 (5.66)	16 (10.06)
Skin/soft tissue infection	11 (6.92)	8 (5.03)
Sepsis	7 (4.4)	8 (5.03)
Catheter infection	2 (1.26)	1 (0.63)
Intra abdominal infection	1 (0.63)	2 (1.26)
Clostridium difficileInfection	0 (0.00)	2 (1.26)
Meningitis	0 (0.00)	1 (0.63)
Unknown	4 (2.52)	7 (4.4)

SD: standard deviation, ICU: intensive care unit

Overall, 55 of 77 patients (71.43%) had microbial culture order before the first dose of vancomycin and the culture results were available only in 28 patients (50.91%). Infusion related adverse drug reactions were detected in 17 patients (22.08%), while the other 60 patients (77.92%) did not show any adverse reactions to vancomycin infusion.

***Post intervention data***

A total of 82 patients were evaluated during a 6 month intervention period. Fever and neutropenia were the most common cause of vancomycin use (15.09%). Compliance with guidelines improved from 38.96% in the pre-intervention period to 59.76% in the post intervention period (*P *= 0.009). No significant improvement was observed for appropriate duration and dosing regimen of vancomycin use (*P *= 0.54 and *P *= 0.15). Only 49 out of 82 patients initiated the treatment correctly. Eleven patients were receiving inappropriate dosage of vancomycin and against the medical advice, one of them had a prior discharge from the hospital,refusing further treatments by signing a consent.Discussion with the physician resulted in the discontinuation of the drug in 50% of the remaining cases (five out of ten) and vancomycin dosage was adjusted in 30.77% of the patients after the second intervention (Table 3).

**Table 3:** Appropriateness of initiation, duration and dosing regimen of vancomycin therapy

Evaluated paremeter	N (and %)	p*-*value
Initiation of vancomycin therapy		
Appropriate initiation before the intervention	30(38.96)	0.009
Appropriate initiation after the intervention	49(59.76)	
Vancomycin therapy stopped following discussions with the physicians	18(54.55)	
Duration of vancomyc in therapy		N/A
Appropriate duration before the intervention	25(83.33)^a^	
Appropriate duration after the intervention	38(77.55)^b^	0.54
Vancomycin therapy stopped following discussions with the physicians	5(50)	
Dosing regimen of vancomycin therapy		
Appropriate dosing regimen before the intervention	42(54.55)	0.5
Appropriate dosing regimen after the intervention	54(65.85)	N/A
Dosing regimen adjusted following discussions with the physicians	8(30.77)	

NA: Not Applicable

a, b The duration of vancomycin therapy was evaluated in patients to whom vancomycin was prescribed and initiated correctly (30 patients before the intervention, and 49 patients after the intervention). The duration of treatment was appropriate in 25 and 38 patients respectively.

From necessary pre-treatment laboratory tests, only complete blood count (CBC) test orders raised significantly (*P *= 0.04) (Table 4). Available temperature charts also showed a significant improvement with a p-value of 0.02 (Table 5). Culture orders before initiation of vancomycin therapy did not improve statistically (*P *= 0.55). However during the intervention period, culture results were significantly raised from 50.91% to 74.19% (*P *= 0.009) (Table 6).

Infusion related adverse drug reactions occurred in 9 patients (10.98%) which compared with 22.08% at the baseline did not change significantly (*P *= 0.06), despite that in 100% of cases these reactions were resolved after notifying the nursing team during the second intervention.

**Table 4:** Assessment of pre-treatment laboratory tests

Laboratory test	Before intervention: n (and %)	After intervention: n (and %)	p-value
CBC test Renal function UA	69(89.61) 64(83.12) 23(23.87)	80(97.56) 73(89.02) 34(41.46)	0.04 0.28 0.13

CBC: complete blood count, UA: urinalysis

**Table 5:** Evaluation of patients after the initiation of vancomycin therapy

Descriptio	Before intervention: n (and %)	After intervention: n (and %)	p-value
Temperature charts Hearing Tests Periodic WBC count monitoring Periodic monitoring of renal function	72(93.51) 0(0.00) 73(94.80) 66(85.71)	82(100) 3(8.82) 82(100) 75(91.46)	0.02 0.54 0.053 0.25

WBC: white blood cell

**Table 6:** Analysis of cultures obtained from 159 patients receiving vancomycin at Taleghani Teaching Hospital.

Description	Before intervention: n (and %)	After intervention: n (and %)	p-value
Culture orders before initiation of therapy Appropriate time of performing culture tests Available results of culture tests in patient notes	55(71.43) 37(67.27) 28(50.91)	62(75.61) 43(69.35) 46(74.19)	0.55 0.81 0.009

Overuse of antibiotics is considered a challenging issue in community and hospital settings (24, 25, 26).Inappropriate use of antibiotics can results in emergence of bacterial resistance (27), which could further affect the patient’s outcomes (28). Thus several studies have been conducted to control and restrict the use of these drugs (27, 29-31).The aim of our study was to control and improve vancomycin use by pharmacist intervention based on HICPAC and IDSA guidelines.

At the baseline 61.04% of vancomycin indications and 16.67% of prescription durations were considered appropriate. Following the interventions performed by a pharmacist inappropriate initiation of vancomycin decreased to 40.24%, but no significant change was observed in inappropriate duration of vancomycin therapy.

In a prospective two-phase study performed by Misan *et al .* (32), the role of educational interventions on vancomycin use was assessed in a large metropolitan teaching hospital. Interventions seemed to have no effects on reducing inappropriate vancomycin prescribing. The study demonstrated that directly consulting with prescribers was the most effective strategy.

In another veterans affairs-affiliated medical center, vancomycin orders were evaluated by Lipsky *et al *. (33), first at baseline and following administrative and educational interventions. Administrative interventions included discussion sessions held by a clinical pharmacist or the chair of the infection control committee who revised routine perioperative prophylaxis orders. Educational interventions consisted of discussions with physicians regarding VRE and appropriate prescribing of vancomycin. Despite a transient decrease after educational interventions, inappropriate use of vancomycin declined from 70% of orders at baseline to 40% after administrative interventions. In accordance with our study, the goal was to assess and promote vancomycin use. However the way that interventions were performed was different. In our study the use of vancomyin was assessed under the supervision of a clinical pharmacist, while Lipsky *et al.,* evaluated its use after direct interventions performed by a clinical pharmacist and the chair of the infection control committee.

In the assessment of vancomycin use conducted by Hamilton *et al.* (34), the effectiveness of pharmacist interventions was confirmed. In this study, the use of vancomycin was evaluated based on guidelines published by CDC through a survey tool. Data collection and primary interventions (such as contacting the physicians in case of non-guideline-adherent treatments) were performed by pharmacists. If inappropriate vancomycin use still continued, a consultation was offered by one of the infectious diseases consultants. Contrary to our study, a hospital-wide education was also provided by the Infection Control Department. At the end, accordance with guidelines for empiric use of vancomycin improved in all categories from 47% in the pre-intervention period to 73% after the intervention (*P *= 0.16).

Similarly, a survey performed by Guglielmo*et al.* used a series of interventions which consisted of automatic 72 h stop orders to improve vancomycin prescribing (35). First, vancomycin orders were reviewed based on HICPAC recommendations, then, series of interventions including an antibiotic 72 h stop order were undertaken. The study demonstrated that inappropriate vancomycin use decreased significantly in the febrile neutropenia patients (*P *= 0.013) and in the patients continuing empirical vancomycin in the absence of gram-positive infection (*P *= 0.002). Conversely, Bolon *et al.* reported that an antibiotic order form intervention did not improve or reduce vancomycin use (36).

Opposite to other studies in which surgical prophylaxis was the most common form of inappropriate use, no case of surgical prophylaxis was observed in our study and empirical therapy remained the major cause of inappropriate vancomycin use (37, 38). The evaluation of vancomycin use in only two specific wards (hematology-oncology and ICU) could possibly explain the lack of surgical prophylactic use in the present study.

Although there was a significant improvement in appropriate initiation of vancomycin, the duration of empirical therapy did not change after intervention of the pharmacist.In a large number of cases empiric treatment continued despite negative culture results. This might demonstrate the physician’s lack of confidence in the laboratory results and thus relies mainly on clinical findings rather than laboratory data. No significant increase of culture orders before initiation of therapy could also confirm this issue, whereby vancomyin was mostly prescribed in the absence of culture orders without taking into consideration the pharmacist’s reminds. Since blood culture information determines the need for vancomycin therapy or proposes another antimicrobial treatment based on susceptibility data, hospital staff must be more educated about the importance and necessity of performing culture tests.

CBC tests, as well as temperature, are considered two main factors determining the length of vancomycin therapy in febrile neutropenic patients. Among patients receiving vancomycin, in our study, a larger number were hospitalized with fever and neutropenia. After the interventions, the physicians wanted to monitor these two factors more closely than before. Pre-treatment CBC tests and temperature charts improved significantly, while periodical monitoring of WBC counts increased from 94.8% to 100%, which was not significant.

In our study like other studies (39, 40), renal function was monitored and the vancomycin dosage was corrected according to the renal function. However, the results of the intervention demonstrated no significant improvement in appropriate dosing regimen. Despite the interventions, in many cases physicians tended to follow routine and prescribe vanomycin as a fixed dose of 1 gram per patient, regardless of the weight. Unlike other studies (41, 42), no therapeutic vancomycin level monitoring was performed in the current study and adjustments of dosage was done only with respect to the renal function.

Based on our study, we suggest additional strategies to improve vancomycin use at Taleghani Teaching Hospital which include:

1. Inform clinicians about appropriate vancomycin use via brochures, handouts, mails and posters.

2. Implementing restrictive measures to control vancomycin use.

3. Direct intervention of clinical pharmacists and infectious diseases consultants to improve awareness of vancomycin usage in order to prevent and control vancomycin resistance.

4. Continue the vancomycin use evaluation to ensure the efficacy of the interventions and also compare the effects of each intervention with the other.

As the first vancomycin use evaluation at Taleghani Teaching Hospital, our study has identified the main factors associated with the inappropriate use of vancomycin in two specific wards with more vancomycin administration. However, the collaboration of physicians, infectious diseases consultants, nurses and direct supervision of clinical pharmacists is required to make the whole intervention more effective.
